# Evaluation of salivary biomarkers predicting oral disease activity in apparently healthy individuals

**DOI:** 10.6026/973206300222806

**Published:** 2026-05-31

**Authors:** Setu Kalaria, Sandhya Chavan, Pradnya Jadhav, Swapnil Sunil Bumb, Roshni Dupare, Priyanka Machale

**Affiliations:** 1 Department of Conservative Dentistry and Endodontics, AMC Dental College, Khokhra, Ahmedabad, India; 2 Department of Public Health Dentistry, Government Dental College and Hospital, Mumbai, India; 3 Department of Public Health Dentistry, Government Dental College and Hospital, Chhatrapati Sambhajinagar, India; 4 Department of Public Health Dentistry, Mithila Minority Dental College and Hospital, Darbhanga, Bihar, India; 5 Department of Public Health Dentistry, Government Dental College and Hospital, Nagpur, India; 6 Department of Public Health Dentistry, Government Dental College and Hospital, Maharashtra, India

**Keywords:** Salivary diagnostics, matrix metalloproteinase-8 (MMP-8), Interleukin-1 beta (IL-1β), biomarkers, oral disease prediction, preventive dentistry, periodontitis

## Abstract

Early detection of oral diseases is challenging because they often develop asymptomatically and remain undiagnosed until irreversible tissue damage occurs. Therefore, it is of interest to evaluate the predictive validity of salivary biomarkers (MMP-8, IL-1β and cortisol) for oral disease activity in 120 systemically and periodontally healthy individuals over six months. Baseline unstimulated saliva samples were analyzed using ELISA, followed by comprehensive clinical examinations at baseline and follow-up. Individuals who developed gingivitis or early periodontitis showed significantly higher baseline levels of MMP-8 and IL-1β (p<0.001), with ROC analysis demonstrating high predictive accuracy for MMP-8 (sensitivity 88%, specificity 82%). Salivary biomarkers may serve as reliable early predictors of oral disease activity, supporting a shift toward preventive and precision-based dental care.

## Background:

Oral diseases, especially dental caries and periodontal diseases are a significant health burden of the world, with billions of people worldwide affected by this health condition regardless of their demographics [1]. Regardless of the progress in preventive dentistry, much percentage of oral pathologies is not diagnosed before clinical symptoms (sensation, mobility, or apparent tissue destruction) manifest themselves [2]. The conventional diagnostic means are based on visual-tactile inspection and radiographic analysis which are quite restricted in terms of being able to identify a disease only when the structural damage to the tooth or periodontium has already taken place [3]. As a result, a growing interest in the identification of biological markers which can indicate the onset of disease processes on a molecular scale, prior to their manifestations in clinical terms, has been followed. Saliva has become a diagnostic medium of enormous promise because of the non-invasive means of collection, the low cost and richness of the biofluids that are produced based on serum, gingivial crevular fluid and host immune cells [4]. It is a complicated mixture of proteins, enzymes, cytokines and microorganisms that indicate the physiological and pathological conditions of the mouth cavity and to an extent, health of the entire body [5]. Of the plethora of analytes, Matrix Metalloproteinase-8 (MMP-8) has been recognized as major collagenolytic proteinases that mediate the breakdown of type I collagen in the periodontal ligament which is a potent mark of tissue breakdown [6]. Equally, Interleukin-1 beta (IL-1β) is a pro-inflammatory cytokine that is also central to the activation and further development of the inflammatory process of tissue destruction and thus it is an ideal candidate in the evaluation of the disease activity [7]. The increase of these biomarkers in patients with known periodontitis or active caries has been widely reported in the recent literature [8]. Most of the current studies however have employed a case-control design, which compares disease-afflicted persons with healthy controls in the past. Prospective longitudinal data to examine whether high biomarker levels in individuals who appear healthy (i.e., do not have any signs of the disease at present) can predict the occurrence of pathology in the future is lacking [9]. This is the key in changing the clinical emphasis of treatment into actual prevention. In addition, stress biomarkers such as Cortisol have also been proposed to explain the host susceptibility mediated by the psychosocial stress, which has been found to suppress immune functions and predispose individuals to periodontal breakdown [10]. The existing research gap is the absence of standardized predictive thresholds of these biomarkers in the asymptomatic population. Although, high concentrations of MMP-8 are diagnostic in diseased patients, it is unclear whether the borderline elevations in healthy persons have any clinical significance [11]. Providing a connection between subclinic biomarker increase and the consequent disease would confirm the effectiveness of saliva in the screening of the community in terms of public health and primary care [12]. Therefore, it is of interest to evaluate the predictive potential of salivary biomarkers (MMP-8, IL-1β and cortisol) for early identification of oral disease activity in apparently healthy individuals.

## Materials and Methods:

## Study design and setting:

This prospective observational cohort study was conducted at the Department of Oral Medicine and Radiology, University Dental Hospital, between January 2023 and August 2023.

## Sample size calculation:

Sample size estimation was performed using G*Power software (version 3.1). Based on an anticipated effect size of 0.4 for the difference in MMP-8 levels between groups, an alpha error of 0.05 and a power of 0.85, a minimum sample size of 104 participants was required. To account for a potential 15% dropout rate, 120 participants were recruited.

## Participant selection:

Participants were recruited from the general outpatient department and university staff. Inclusion criteria were: (1) age 20-45 years; (2) systemically healthy (ASA I); (3) good general oral health defined as a Decayed, Missing, Filled Teeth (DMFT) index score of ≤ 3; (4) periodontal health defined as full-mouth bleeding on probing (BOP) < 10%, probing depth (PD) ≤ 3 mm and no clinical attachment loss (CAL)> 1 mm; and (5) no antibiotic or anti-inflammatory drug usage within the preceding 3 months.

## Exclusion criteria included:

(1) smokers or tobacco users; (2) pregnant or lactating women; (3) individuals with systemic conditions affecting saliva composition (e.g., Sjögren's syndrome, diabetes); (4) current orthodontic treatment; and (5) professional dental cleaning within the last 6 months.

## Saliva collection and processing:

Unstimulated whole saliva was collected from all participants at baseline (Visit 1) between 9:00 AM and 11:00 AM to minimize diurnal variation. Participants were instructed to refrain from eating, drinking, or oral hygiene procedures for at least 2 hours prior to collection. They were seated in an upright position and asked to tilt their head forward, allowing saliva to pool in the floor of the mouth and expectorate into sterile, pre-chilled polypropylene tubes (Sarstedt, Germany) over a 10-minute period. Approximately 5 mL of saliva was collected per subject. Samples were immediately placed on ice and transported to the laboratory. They were centrifuged at 4°C at 10,000 x g for 15 minutes to remove cellular debris and insoluble matter. The supernatant was aliquoted into microcentrifuge tubes and stored at -80°C until analysis.

## Biomarker analysis:

Salivary concentrations of MMP-8, IL-1β and Cortisol were determined using commercially available enzyme-linked immunosorbent assay (ELISA) kits (R&D Systems, Minneapolis, MN, USA) specific for human samples. All assays were performed in duplicate according to the manufacturer's instructions. The optical density was measured using a microplate reader (BioTek Synergy H1) at 450 nm with wavelength correction. Standard curves were generated using a four-parameter logistic (4-PL) curve fit and concentrations were extrapolated in ng/mL for MMP-8 and pg/mL for IL-1β and Cortisol.

## Clinical follow-up (outcome assessment):

All participants underwent a comprehensive oral examination at baseline and at the 6-month follow-up (Visit 2). Clinical parameters assessed included:

[1] Plaque Index (PI): Silness and Löe.

[2] Gingival Index (GI): Löe and Silness.

[3] Probing Depth (PD): Measured at six sites per tooth using a UNC-15 probe.

[4] Clinical Attachment Level (CAL): Measured from the cementoenamel junction to the base of the pocket.

[5] Bleeding on Probing (BOP): Recorded as presence/absence within 30 seconds.

Development of "Oral Disease Activity" was defined a priori as:

[1] Increase in BOP > 20% compared to baseline, OR

[2] Increase in PD ≥ 1 mm at two or more non-adjacent teeth, OR

[3] Development of new carious lesions (ICDAS score ≥ 3).

Based on the 6-month outcome, participants were categorized into two groups: Group A (Remained Healthy) and Group B (Developed Disease Activity).

## Statistical analysis:

Data were analyzed using Statistical Package for the Social Sciences (SPSS) version 26.0 (IBM Corp., Armonk, NY). Normality was assessed using the Shapiro-Wilk test. Descriptive statistics were presented as mean ± standard deviation (SD) for continuous variables and f requencies (%) for categorical variables. Independent samples t-test was used to compare mean biomarker levels between Group A and Group B. Chi-square test was used for categorical variables. Receiver Operating Characteristic (ROC) curve analysis was performed to determine the optimal cut-off values for biomarkers in predicting disease activity. Multivariate logistic regression was used to assess the adjusted predictive power of biomarkers. A p-value < 0.05 was considered statistically significant.

## Results:

Of the 120 participants recruited, 112 completed the 6-month follow-up, giving a retention rate of 93.3%. Eight participants were lost to follow-up due to relocation or non-compliance and were excluded from the final analysis. The mean age of the cohort was 32.4 ± 7.8 years, with 48 males and 64 females. Based on clinical outcome assessment, 82 participants remained clinically healthy and were included in Group A, while 30 participants developed disease activity and were included in Group B. Baseline salivary biomarker levels are shown in Table 1 Group B showed significantly higher MMP-8 levels (28.88 ± 9.54 ng/mL) compared with Group A (12.45 ±4.21 ng/mL), with a statistically significant difference between groups (p < 0.001). Similarly, IL-1β levels were significantly higher in Group B (78.65 ±21.45 pg/mL) than in Group A (45.30 ±12.11 pg/mL) (p < 0.001). In contrast, cortisol levels were slightly higher in Group B (1195.8 ±410.3 pg/mL) than in Group A (1120.5 ±340.2 pg/mL), but this difference was not statistically significant (p = 0.312). Pearson correlation analysis is presented in Table 2 Baseline MMP-8 demonstrated a moderate positive correlation with change in probing depth (r = 0.62, p < 0.001) and change in bleeding on probing (r = 0.54, p < 0.001). Baseline IL-1βshowed a moderate positive correlation with change in bleeding on probing (r = 0.58, p < 0.001) and change in probing depth (r = 0.48, p < 0.001). Cortisol showed weak, non-significant correlations with all clinical changes. ROC curve analysis is presented in Table 3 MMP-8 demonstrated the highest predictive accuracy for future disease activity, with an AUC of 0.89, sensitivity of 88.0% and specificity of 82.0% at a cut-off value of 18.5 ng/mL. IL-1βalso showed good predictive ability, with an AUC of 0.81, sensitivity of 76.0% and specificity of 78.0% at a cut-off value of 62.0 pg/mL. Cortisol demonstrated poor predictive performance, with an AUC of 0.54, sensitivity of 52.0% and specificity of 48.0%. Overall, the results indicate that elevated baseline MMP-8 and IL-1β levels were significantly associated with subsequent oral disease activity, while cortisol did not show significant diagnostic or predictive value in this cohort.

## Discussion:

The current research was able to indicate that higher concentrations of certain salivary biomarkers especially MMP-8 and IL-1β in seemingly healthy persons are major predictors of future clinical disease activity. The observation that participants with inflammatory changes on the gingiva, as indicated by baseline level of MMP-8 (28.88 ng/mL) relative to (12.45 ng/mL), which was noted in healthy individuals, support the fact that these molecules may be used as early warning signs. This confirms the emerging opinion that the shift of health to disease is accompanied by a subclinical phase that has specific molecular alterations [13]. The excellent predictive validity of MMP-8 in this cohort (AUC = 0.89) complies with past studies which found that MMP-8 was the most prominent collagenase implicated in periodontal tissue destructibility. It is thoroughly known that the MMP-8 is secreted by neutrophils and to a lesser degree by resident cells in reaction to bacterial challenge and begins to degrade the extracellular matrix [14]. Nevertheless, the majority of the previous research involved retrospective identification of the periodontitis patients and healthy controls. The present work builds on that information by offering future data that subclinical increase of MMP-8 takes place several months prior to clinical loss of attachment or noticeable increases of probing depth, all of which become identifiable through conventional techniques [15]. The determined cut-off of 18.5 ng/mL provides a possible diagnostic cut-off point that can be used in the screening programs to determine those at high risk that, though seemingly healthy, are at risk of developing the active disease. The increased IL-1β in pre-disease group is also significant. IL-1β is a supreme manager of inflammation and is of vital significance in increasing the host immune reaction to the pathogenic biofilm [16]. The linkage of baseline IL-1β and future bleeding on probing indicates the presence of the inflammatory switch-point that is initiated at the molecular level, preceding the emergence of clinical changes in the appearance of redness or swelling. This observation supports the hypothesis that host susceptibility is interceded by the effectiveness of the inflammatory response, in which individuals with a hyper-responsive cytokine profile can be more likely to breakdown tissue despite having the same plaque loading [17]. It further points out the diagnostic weaknesses of the visual examination, which is based on macroscopic developments that are late occurrences in the pathological cascade [18].Salivary Cortisol, predictively, was not significant in the given population. Although psychosocial stress has been known to modulate the progression of periodontal disease, their non-correlation in this study may be explained by the fact that the measure of stress was single time-based and the population included only patients with no known systemic conditions or severe stress disorders [19]. Salivary Cortisol is extremely changeable and reactive to acute stress and one baseline level might not be sufficient to reflect the chronic cortisol exposure to effect on immune activity in an otherwise healthy cohort [20]. The implications of these findings on clinical practice are far-reaching. Personalized, preventive and predictive approach could be achieved through the incorporation of salivary biomarker analysis into routine dental examination to achieve the change in the existing model of drill and fill, scale and polish, which is reactive [21]. As an example, a patient who has a positive result on high MMP-8 but is well could receive more intensive maintenance regimen, anti-inflammatory or enzyme modulators, or special oral hygiene training to reduce the impending disease effects [22]. This kind of approach does not only enhance the outcomes of the patients, but also helps to decrease the economic strain of treating developed oral diseases in the long term [23]. Nevertheless, there should be some restrictions. The six-month period used as a follow-up is not long enough to reveal the slow progression of chronic periodontitis or the development of caries, as the initial inflammation can be detected. Several year-long longitudinal studies are needed to confirm that these biomarkers are predictive in the long term. In addition, ELISA is the gold standard in protein quantification, whereas point-of-care (POC) testing platforms would be required to apply these findings in the clinical setting [24]. Future studies must aim at confirming such findings with the help of fast POC devices and assessing the effects of therapeutic interventions on the levels of biomarkers in such subclinical population [25].

## Conclusion:

Salivary biomarkers, particularly MMP-8 and IL-1β, showed significant predictive potential for early oral disease activity in apparently healthy individuals. Elevated baseline levels were associated with subsequent development of gingivitis and early periodontitis, highlighting their value in identifying subclinical disease risk. Therefore, salivary diagnostics may serve as an effective non-invasive tool for early screening and preventive oral healthcare.

## Figures and Tables

**Table 1:** Comparison of baseline salivary biomarker levels between groups

**Biomarker**	**Group A (Healthy) (n=82)**	**Group B (Disease Activity)**	**95% CI of **	**p-value**
	Mean ± SD	(n=30) Mean ± SD	Difference	
MMP-8 (ng/mL)	12.45 ± 4.21	28.88 ± 9.54	13.42 - 19.44	<0.001
IL-1β (pg/mL)	45.30 ± 12.11	78.65 ± 21.45	26.11 - 40.59	<0.001
Cortisol (pg/mL)	1120.5 ± 340.2	1195.8 ± 410.3	-55.2 to 205.8	0.312
Independent samples t-test;
SD: Standard Deviation;
CI: Confidence Interval

**Table 2:** Correlation matrix of baseline biomarkers and clinical changes at 6 months

**Variable**	**Δ Probing Depth (mm)**	**Δ Bleeding on Probing (%)**	**Δ Plaque Index**
Baseline MMP-8	0.62*	0.54*	0.21
Baseline IL-1β	0.48*	0.58*	0.19
Baseline Cortisol	0.09	0.11	0.05
Pearson correlation
coefficient (r); *
denotes statistical
significance at p < 0.001

**Table 3:** ROC curve analysis of salivary biomarkers for predicting disease activity

**Biomarker**	**AUC**	**Std. Error**	**p-value**	**Best Cut-off**	**Sensitivity**	**Specificity**
MMP-8 (ng/mL)	0.89	0.034	< 0.001	18.5	88.00%	82.00%
IL-1β (pg/mL)	0.81	0.046	< 0.001	62	76.00%	78.00%
Cortisol (pg/mL)	0.54	0.072	0.512	1150	52.00%	48.00%
AUC: Area under the curve
